# Investigation of severe dengue outbreak in Maumere, East Nusa Tenggara, Indonesia: Clinical, serological, and virological features

**DOI:** 10.1371/journal.pone.0317854

**Published:** 2025-02-18

**Authors:** Marsha S. Santoso, Mario B. R. Nara, Dwi Kurniawan Nugroho, Benediktus Yohan, Asep Purnama, Angela M. B. Boro, Rahma F. Hayati, Erlinda P. Gae, Dionisius Denis, Bunga Rana, Martin L. Hibberd, R. Tedjo Sasmono

**Affiliations:** 1 Eijkman Research Center for Molecular Biology, National Research and Innovation Agency, West Java, Indonesia; 2 Exeins Health Initiative, Jakarta, Indonesia; 3 TC Hillers Hospital, Maumere, Sikka, East Nusa Tenggara, Indonesia; 4 Department of Infection Biology, London School of Hygiene and Tropical Medicine, London, United Kingdom; Institute of Tropical Medicine: Instituut voor Tropische Geneeskunde, BELGIUM

## Abstract

**Background:**

Dengue, an acute febrile disease caused by dengue virus (DENV) infection, is endemic to Indonesia. During early 2020, an outbreak of severe dengue occurred in Maumere, East Nusa Tenggara province, a region with low dengue endemicity with limited data on the characteristics of the circulating DENV. By 18 March 2020, 1396 cases were reported with 14 fatalities. Investigation was conducted to understand the cause and characteristics of the outbreak.

**Methods:**

Sera were collected from 133 patients with dengue-like symptoms through random sampling at TC Hillers Hospital, Maumere during outbreak between February and June 2020. Dengue was confirmed using NS1 and/or RT-PCR detection. Serological status was determined using IgG/IgM ELISA and plaque reduction neutralization test (PRNT). DENV serotyping and genome sequencing were performed to identify the DENV serotype and genotype.

**Results:**

We recruited suspected dengue patients attending the hospital during the outbreak. Dengue was confirmed in 72.2% (96/133), while 18.8% (25/133) were diagnosed as probable dengue. Children under 18 years old accounted for 85.1% (103/121) of dengue cases. Severe dengue accounted for 94.2% (81/86) of cases. Secondary infections made up 92.6% (112/121) of cases. Serotyping detected 87.3% (62/71) as DENV-3, 7.0% (5/71) as DENV-4, 2.8% (2/71) as DENV-1, and 2.8% (2/71) as DENV-2. Phylogenetic analysis revealed close evolutionary relationship of Maumere DENV to viruses from other Indonesian regions, especially Bali and Kupang. PRNT on DENV-3 secondary infections patients detected the presence of DENV-2 and DENV-4 neutralizing antibodies.

**Conclusion:**

The severe dengue outbreak in Maumere is caused by DENV-3 introduced from nearby islands. The high proportion of secondary infections likely contributes to the severity of the disease. The high percentage of anti-dengue neutralizing antibodies for multiple serotypes and the high proportion of anti-dengue IgG in young children suggests a history of dengue transmission with a high infection rate in the area.

## Introduction

Dengue is a viral infection caused by dengue virus (DENV) with an estimated global burden of 50 million infections every year [1]. Dengue can be asymptomatic, mildly symptomatic as dengue fever (DF), more severe with signs of plasma leakage as dengue hemorrhagic fever (DHF), or progress into circulatory failure as dengue shock syndrome (DSS) [2]. Dengue outbreaks develop quickly and require immediate action to control and reduce transmission. Outbreak investigations are important in order to control the health problem, to increase understanding of the disease characteristics and transmission dynamics, and to guide public health programs, policies, and legislations [3]. In DENV-endemic countries, the main goal is to minimize the clinical and socioeconomic impact of dengue by strengthening control measures and mitigating the risk for any future outbreaks [4].

Dengue is endemic to Indonesia, a vast archipelagic country which continues to see cyclical outbreaks throughout the years, with a 2019 national incidence rate of 51.53 per 100 000 population [5]. There are four serotypes of DENV (DENV-1, -2, -3, and -4), all of which have been found co-circulating across the 34 provinces of Indonesia [6]. Virologic, immunologic, and genomic data on dengue in Indonesia are mostly gathered from urban cities in the western region of the country [6] while data from the more rural, eastern regions such as East Nusa Tenggara province are still limited.

East Nusa Tenggara province comprises of over a thousand islands and is known to have low dengue endemicity [7]. The only serotype data available from this region was from a previous study by our group conducted in Kupang, the capital city of the province, in 2016–2017 [8]. Although data from Kupang is available, showing that the predominant DENV serotype circulating the city was DENV-3 [8], it is unknown whether this serotype is also prevalent in other islands in the province. Maumere is another major city in East Nusa Tenggara but located on a different island from Kupang, and has no previous data on DENV serotype circulating in the city. In late 2019, an outbreak of severe dengue was reported in Maumere. In Indonesia, dengue outbreaks are alerted when there is a 50% increase of cases per week compared to the previous year [9]. The outbreak of severe dengue in areas which were previously reported to have a low dengue endemicity warrants investigation. Maumere dengue outbreak has received national public attention because of its magnitude and reported severity and fatalities [10]. In this study, we conducted a dengue outbreak investigation in Maumere to decipher the causative agents and understand the disease dynamics which may be useful to prevent future outbreaks.

## Materials and methods

### Geographical features, dengue incidences, and sample collection of study sites

This study was conducted in TC Hillers District Hospital in Maumere, Sikka, East Nusa Tenggara province, Indonesia. Maumere is the capital of Sikka regency, one of 21 regencies of East Nusa Tenggara province. It is the second largest city on Flores island with a total geographic area of 170 km^2^ and a population of 87 720 people in 2020 [11]. Maumere is 370 km away from the Democratic Republic of Timor-Leste, which shares a land border with the West Timor regencies of East Nusa Tenggara province. Maumere has a tropical climate with rainy season from December to April.

**Fig 1:**
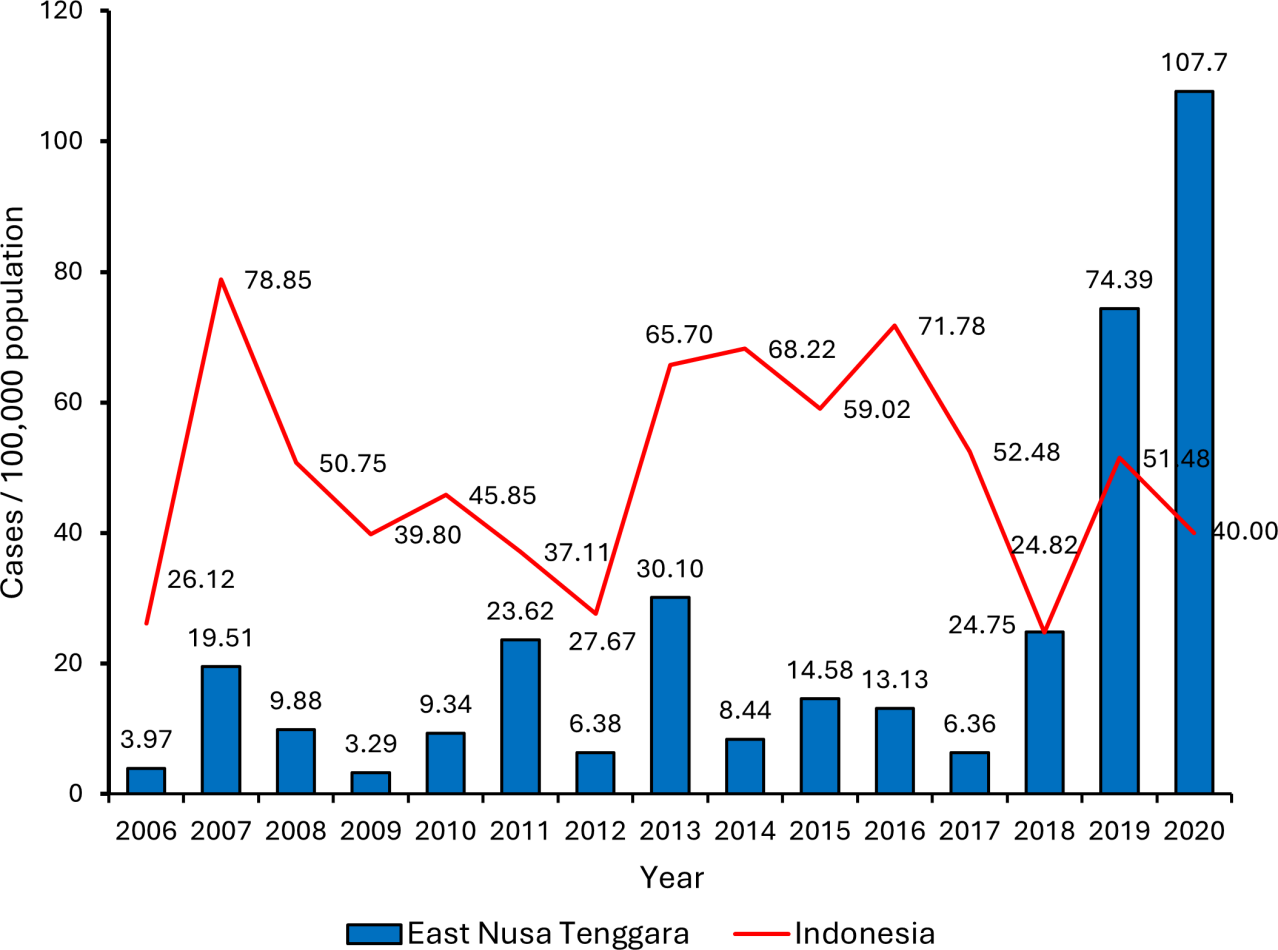
Incidence rate of dengue in East Nusa Tenggara province compared to national incidence rate of dengue in Indonesia.

Prior to 2017, the incidence rate of dengue in East Nusa Tenggara are relatively low compared to other provinces, suggesting low endemicity [7]. However, since 2018, dengue cases more than tripled each year, then in 2020, the incidence rate drastically increased to more than twice the national dengue incidence rate (Fig 1) [12]. The Sikka District Health Office declared the situation as dengue outbreak when Sikka regency reported 1216 cases, the highest number of cases out of the six regencies in East Nusa Tenggara that declared dengue outbreaks [13].

Sample size was determined using the sample calculation for cross-sectional observational studies, with an expected prevalence of dengue cases among febrile patients as 60% and precision of 10%, resulting in a minimum sample size of 93. Taking into consideration the possibility of dropouts and unusable samples with a rate of 10%, the minimum number of samples targeted is 102 samples.

In this study, sera were collected from 133 participants recruited by five on-site investigators/nurses through random sampling with inclusion criteria of acutely febrile patients between the ages of 6 months until 60 years old, both male and female, having fever above 38^o^C for less than 5 days with dengue-like symptoms attending the hospital from 6^th^ February to 9^th^ June 2020, coinciding with the city’s rainy season. As according to the WHO-SEARO Guidelines on Dengue, dengue-like symptoms were defined as acute febrile illness with two or more of the following symptoms: headache, retro-orbital pain, myalgia, arthralgia, rash, haemorrhagic manifestations, leucopenia (white blood count ≤  5000 cells/mm^3^), thrombocytopenia (platelet count <  150 000 cells/mm^3^), and rising haematocrit (5–10%) [4]. Patients with clear diagnosis of bacterial, gastrointestinal, pulmonary infections were excluded from the sample.

### Clinical and laboratory examinations for dengue and other arbovirus infections

All the recruited samples were screened for DENV NS1, IgG, and IgM using Standard Q Dengue Duo (SD Biosensor, Korea) rapid tests on site according to manufacturer’s instructions. Other data including patient demographics, clinical signs and symptoms, and basic hematology laboratory results were recorded through questionnaires and medical records. The questionnaire used is adapted from the Case Investigation Form from the WHO-SEARO Comprehensive Guidelines on Dengue. Serum samples were then sent to Eijkman Institute in Jakarta for further testing to confirm dengue and/or other arbovirus infections. Viral extraction was performed using QIAamp Viral RNA Mini Kit (Qiagen, Germany) and DENV nucleic acid detection was performed using Simplexa Dengue qRT-PCR (DiaSorin, Saluggia, Italy). Immunologic status was determined using NovaLisa Dengue Virus IgG ELISA (Novatec Immundiagnostica GmbH, Dietzenbach, Germany) with positive results indicating secondary dengue infections. Samples that were negative for dengue NS1 antigen rapid test and qRT-PCR were further tested for detection of pan-flavivirus and pan-alphavirus families to detect possible infection with other arboviruses such as Zika (ZIKV), Japanese encephalitis (JEV) or chikungunya virus (CHIKV), using methods described previously [14,15].

Samples were classified as virologically confirmed for dengue based on positive NS1 antigen rapid test and/or positive qRT-PCR results according the WHO-SEARO guideline for dengue prevention and control [4]. Samples with negative NS1 rapid test and qRT-PCR results but positive anti-dengue IgG and/or IgM rapid test were classified as probable dengue [4]. Samples with negative NS1, IgG/IgM rapid tests, and qRT-PCR were classified as non-dengue.

Plaque reduction neutralization test (PRNT) to all four DENV serotypes was performed on sixteen samples with secondary infections of the dominant serotype (i.e., DENV-3) to determine the titer of anti-dengue antibody according to the serotype of their past dengue infections. Samples were selected based on sera volume availability remaining after all the previous tests mentioned above, then selected randomly from that group. The age distribution of these selected samples reflected similarly to that of the larger group from which they were selected. The PRNT method performed was based on previously optimized and validated PRNT_50_ assay for the detection of neutralizing antibodies to the four serotypes of DENV [16]. Serum samples were heat-inactivated at 56^o^C and assayed in four separate PRNT runs for each of the four different DENV serotype challenge viruses. The PRNT was performed by incubating a mixture of serially diluted sample sera with a certain virus concentration and inoculating them into seeded well-plates. The cell lines, secondary antibodies, serotype-specific primary antibodies and challenge viruses used in the procedure has been described previously [17].

### Genome analysis and determination of virus genotype

Full length Envelope (E) gene sequences (1485 nt for DENV-1, -2, -4 and 1479 nt for DENV-3) were generated for phylogenetic analyses of DENV from Maumere based on a protocol previously described [18]. Complementary DNA (cDNA) were synthesized by reverse transcription using Superscript III Reverse Transcriptase (Invitrogen-Thermo Scientific, USA) and then amplified with *Pfu* Turbo Polymerase (Stratagene-Agilent Technologies, USA) with primers targeting full E gene coverage. The amplified E genes were then used as template for cycle sequencing reaction using BigDye Dideoxy Terminator kits v.3.1 (Applied Biosystems-Thermo Scientific) and six overlapping primers for each serotype to obtain the full-length envelope gene sequences [18]. Contig mapping was performed using SeqScape v.2.5 software (Applied Biosystems).

Phylogenetic trees for each DENV were inferred from these Maumere sequences and reference strains downloaded from the GenBank database. Datasets incorporating closely related sequences from other Indonesian cities and nearby countries were generated based on initial nucleotide BLAST queries. The geographic location of other Indonesian cities relative to Maumere is depicted in the Supplementary Figure (S1 Fig). Further selection and trimming of resulting taxa to closely-related strains was conducted by generating an initial maximum likelihood tree by minimum evolution method in MEGA X software [19], while additional reference strains were added for genotype presentation and classification. In each DENV serotype alignment, up to 95 taxa were selected for visual clarity of the generated tree.

Each DENV sequence alignment was analyzed using Bayesian Markov Chain Monte Carlo (MCMC) algorithm through BEAUti 2 graphical interface and run in BEAST v.2.6.7 [20]. Selection of the statistical model for likelihood was calculated using MEGA X software [19], resulting in a Tamura-Nei model with four gamma parameters and invariant sites (TN93 +  Γ4 + I) as the best fit for all four DENV alignments. The molecular clock analyses were set using a relaxed clock log normal [21] and a coalescence Bayesian skyline tree prior [22], set to generate 100 million chains sampled for every 1000 chains with initial estimated evolutionary rate of 7.6 ×  10^-4^ substitutions per site per year [23]. The MCMC trace was analyzed using Tracer v.1.7.2 to monitor the adequate Effective Sampling Size (ESS) for all parameters after 10% burn-in. Maximum clade credibility (MCC) tree was created using TreeAnnotator v.2.6.6 and visualized using FigTree v.1.4.4. The classification of the genotypes in each DENV serotype was based on classifications by Goncalvez et al. [24], Twiddy et al. [25], Lanciotti et al. [26], and Lanciotti et al. [27] for DENV-1, -2, -3, and -4, respectively.

All laboratory experiments were performed at the Dengue Research Laboratory at the Eijkman Institute for Molecular Biology, Jakarta, Indonesia. The laboratory is internationally certified for Good Clinical Laboratory Practice. The reporting of the data follows the Strengthening the Reporting of Observational Studies in Epidemiology (STROBE) guidelines [28].

### Statistical analysis

Demographic and clinical data were compared using Pearson’s Chi-square or Kruskal-Wallis tests as appropriate. Binomial regression analyses were also performed to measure effects of potentially relevant clinical covariates such as gender, age, and immunologic status for reported dengue-typical symptoms and disease severity. Dengue attack rate (AR) is calculated based on regression models estimating FOI according to the mean age of primary and secondary infections as described in a previous study [29]. All statistical analyses were performed on R Studio software (http://www.r-project.org) with *p*-values of less than 0.05 as cut-off for statistical significance.

### Ethical approval

The Eijkman Institute Research Ethics Commission (EIREC) reviewed and approved the study protocol with approval No. 113/2017. Written informed consent was collected from all study participants, or from parents or legal guardians on behalf of minors.

## Results

### Patient characteristics, clinical features, and serotype distribution

**Fig 2:**
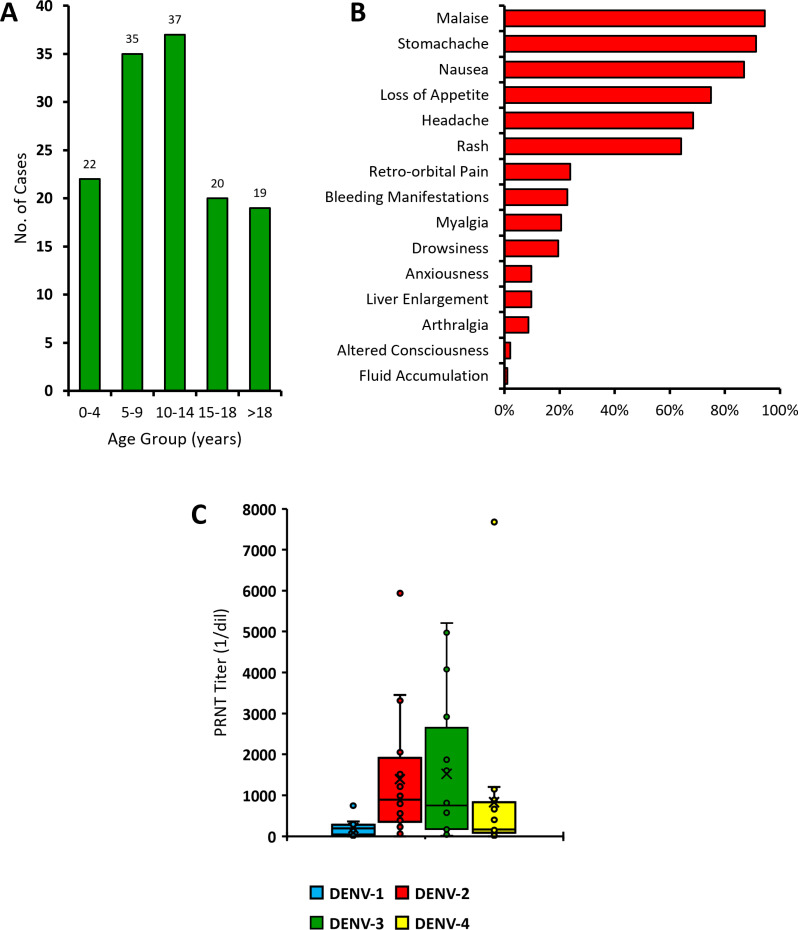
Demographic, clinical, and laboratory characteristics of dengue patients in Maumere. Age distribution of acutely febrile recruited patients (A). Reported symptoms of recruited acutely febrile patients (B). Plaque reduction neutralization test (PRNT) titers to four DENV serotypes on samples with secondary DENV-3 infections (C). Detailed antibody titer for each serotype is shown in S1 Table.

Out of a total of 133 acutely febrile patients with symptoms suggestive of dengue, 72.2% (96/133) were virologically confirmed for dengue through RT-PCR and/or NS1 antigen detection, while 18.8% (25/133) were diagnosed as probable dengue (Table 1). From these confirmed and probable dengue cases, 85.1% (103/121) were children and adolescents under the age of 18, with a median age of 10 years old (IQR =  6–16) (Fig 2A). Aside from fever, the five most reported symptoms were malaise, stomachache, nausea, loss of appetite, and headache (Fig 2B). Out the confirmed and probable dengue cases with clinical data, 94.2% (81/86) were categorized as the more severe forms of dengue (DHF or DSS), while only 5.8% (5/86) were categorized as DF (Table 2).

**Table 1:** Laboratory diagnoses on acutely febrile recruited patients.

Category	Total (N = 133)
*DENV antigen and RNA detection, N (%)*	
RT-PCR-positive	71 (53.4)
RT-PCR-negative, NS1-positive	25 (18.8)
**Virologically confirmed dengue**	**96 (72.2)**
*DENV serotype distribution, N (%)* ^*^	
DENV-1	2 (2.8)
DENV-2	2 (2.8)
DENV-3	62 (87.3)
DENV-4	5 (7.0)
Mixed serotypes	0 (0.0)
*DENV serological diagnosis, N (%)*	
RDT IgM-positive	66 (49.6)
ELISA Indirect IgG-positive	122 (91.7)
**Probable dengue**	**25 (18.8)**
**Non-dengue** (NS1-, RT-PCR-, and IgM-negative)	**12 (9.0)**

**Table 2:** Severity, immunology, and haematology features of confirmed and probable dengue patients.

Parameters	Total (n = 121)
*Severity, N(%)* *	
DF	5 (5.8)
DHF	63 (73.3)
DSS	18 (20.9)
*Infection status, N(%)*	
Primary	9 (7.4)
Secondary	112 (92.6)
*Hematology features, Median (IQR)* *	
Platelet (x10^3^/µL)	30 (13.3–60.8)
Hematocrit (%)	39 (36.4–42.1)
WBC ( × 10^3^/µL)	4.0 (2.7–5.2)

*) Data only available for 86 samples.

DENV nucleic acid was detected in 71 patients. Of these, 87.3% (62/71) were of the dominant serotype DENV-3 (87.3%), 7.0% (5/71) were DENV-4, 2.8% (2/71) were DENV-1, and 2.8% (2/71) were DENV-2. Unfortunately, due to low numbers of samples for DENV-1, -2, and -4, a comparison of clinical features between serotypes could not be performed. Patients that tested negative for DENV were then screened for other arboviruses such as CHIKV, JEV, and ZIKV using RT-PCR with pan-alphavirus and pan-flavivirus primers respectively, but were all negative (Table 1).

### Serological status of selected patients

Serological testing showed that 92.6% (112/121) were secondary infections, which had statistically significantly lower median platelet count (27 ×  10^3^/uL) compared to primary infections (54 ×  10^3^/uL, *p* = 0.045). Over 90% (58/63) of children under 10 years old tested positive for anti-dengue IgG using indirect ELISA, demonstrating a high infection rate of dengue, even in young children.

Out of 56 samples with secondary DENV-3 infections, PRNT was performed on 16 samples. Of these, 88% (14/16) had neutralizing antibody to all four serotypes, 1 sample had neutralizing antibodies to three serotypes (DENV-1, -3, and -4), and 1 sample had neutralizing antibodies to two serotypes (DENV-2 and -3). The median PRNT_50_ titers for DENV-1, -2, -3, and -4 antibodies are 194, 892, 751, and 165, respectively (Fig 2C).

Dengue AR was calculated based on regression models estimating FOI according to the mean age of primary and secondary infections [25]. Based on those models, the predicted AR of dengue in Maumere is higher compared to other regions in Indonesia experiencing dengue outbreaks during the previous year (Table 3).

**Table 3:** Comparison of dengue attack rates according to the mean age of primary and secondary dengue infections in different regions of Indonesia.

Site (Year)	AR (95% CI)	
	Primary infection	Secondary infection
Maumere (2020)	0.28 (0.12–0.63)	0.18 (0.15–0.23)
Jember (2019–2020) [30]	0.06 (0.04–0.19)	0.07 (0.05–0.12)
Tarakan (2018–2019) [31]	0.17 (0.12–0.24)	0.21 (0.14–0.30)
Malinau (2018–2019) [31]	0.09 (0.07–0.12)	0.12 (0.09–0.15)

### Phylogenetic analysis of DENV

**Fig 3:**
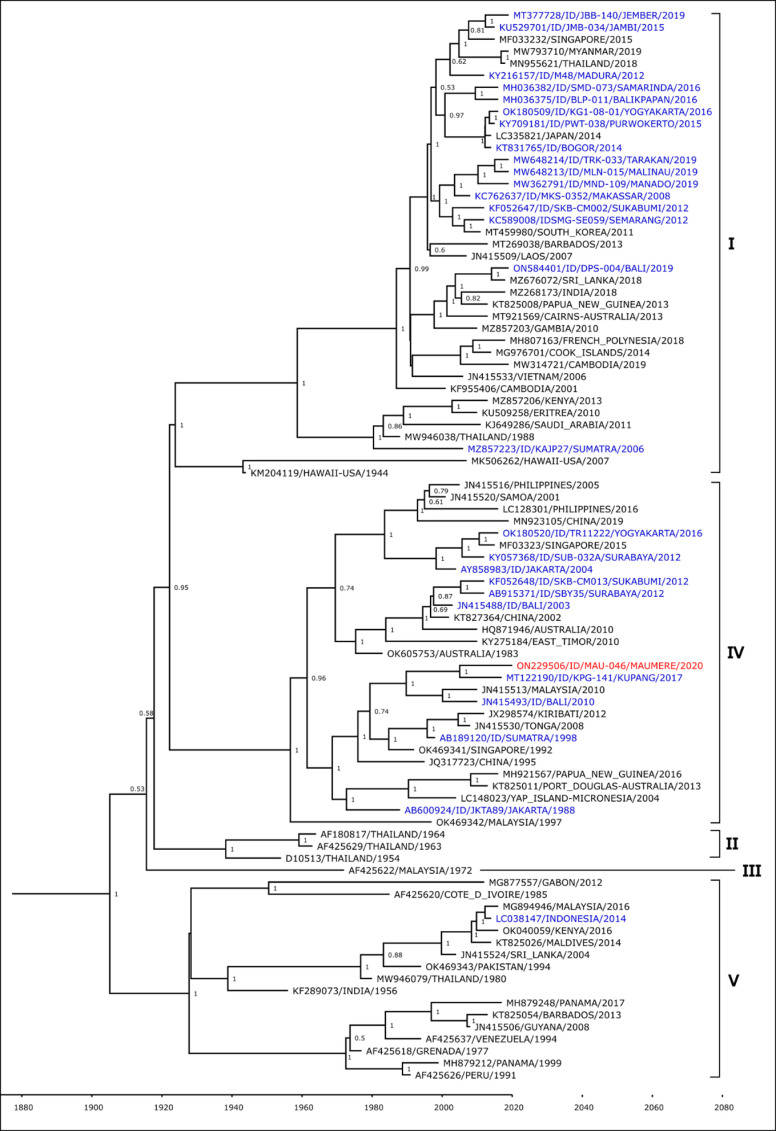
Phylogenetic analysis of Maumere DENV-1 (red font) together with closest strains from Indonesia (blue font) and strains from surrounding countries (black font).

Complete E-gene of 18 DENV isolates from Maumere – one DENV-1, one DENV-2, twelve DENV-3, and four DENV-4 – were successfully sequenced, assembled and analyzed. All sequences have been deposited in GenBank database with accession number ON229523 - ON229506. Through phylogenetic analysis, the DENV-1 isolate was shown to belong to the Genotype IV group based on Goncalvez’s classification [24] (Fig 3). This isolate is closely related to strain from Indonesian province of Bali, isolated in 2010, and Kupang, isolated in 2017.

**Fig 4:**
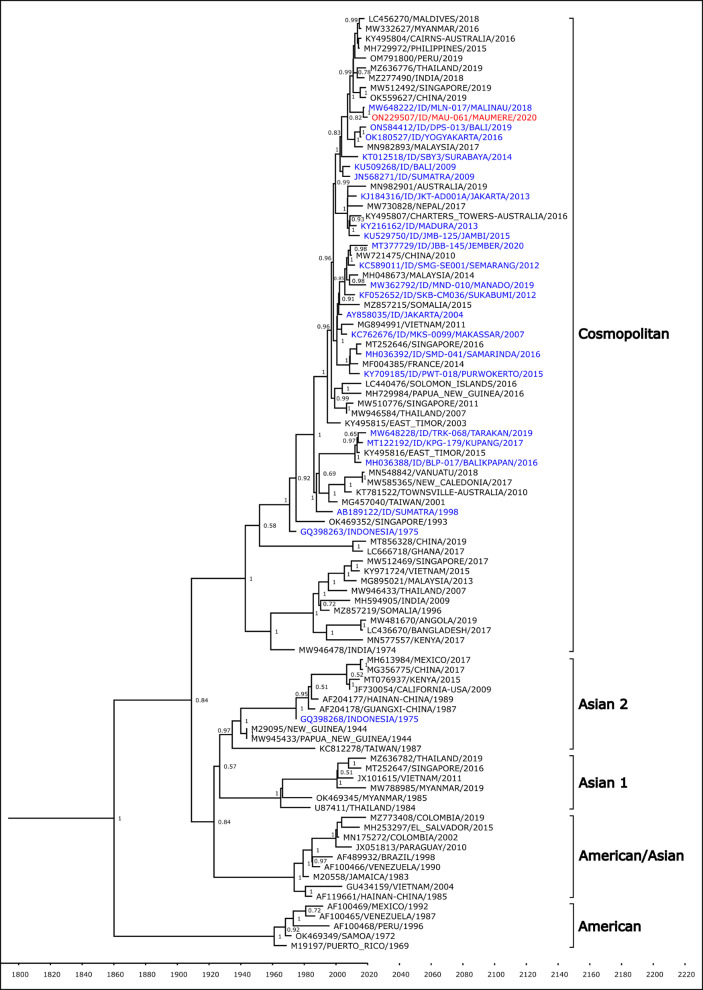
Phylogenetic analysis of Maumere DENV-2 (red font) together with closest strains from Indonesia (blue font) and strains from surrounding countries (black font).

The DENV-2 isolate was shown to belong to the Cosmopolitan genotype based on Twiddy’s classification [25] (Fig 4). The Maumere isolate formed a monophyletic clade with strain from Indonesian cities of Malinau in North Kalimantan, Bali, and Yogyakarta.

**Fig 5:**
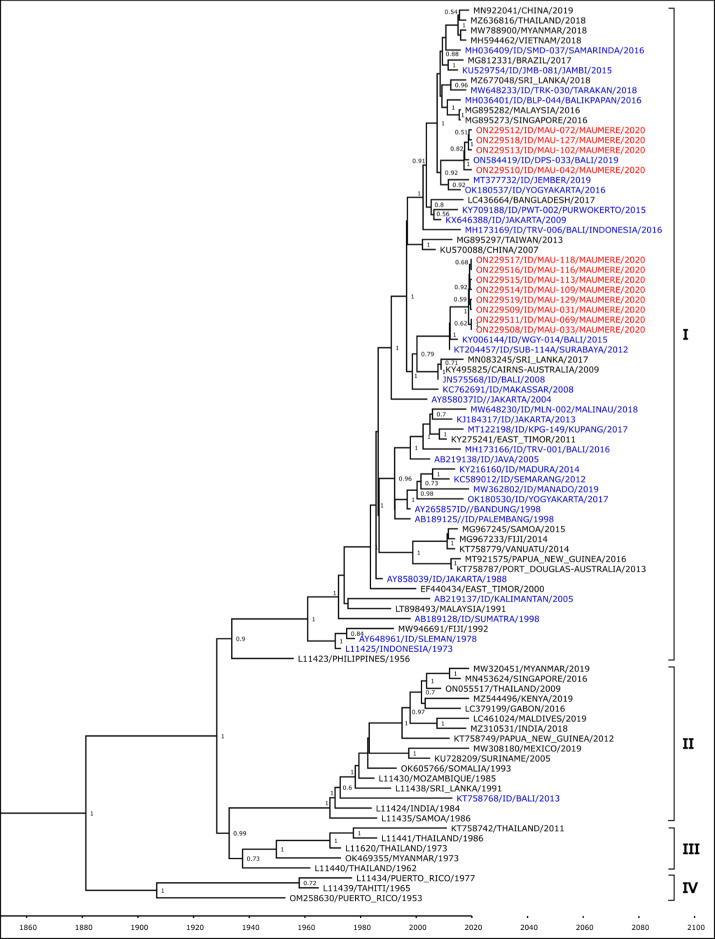
Phylogenetic analysis of Maumere DENV-3 (red font) together with closest strains from Indonesia (blue font) and strains from surrounding countries (black font).

The predominant serotype DENV-3 isolates were shown to belong to Genotype I group based on Lanciotti’s classification [26] (Fig 5). These 12 isolates formed two separate lineages, one lineage is closely related with strains from Indonesia cities in Kalimantan Island (Tarakan, Samarinda, and Balikpapan), while the other lineage is closely related with strains from Bali.

**Fig 6:**
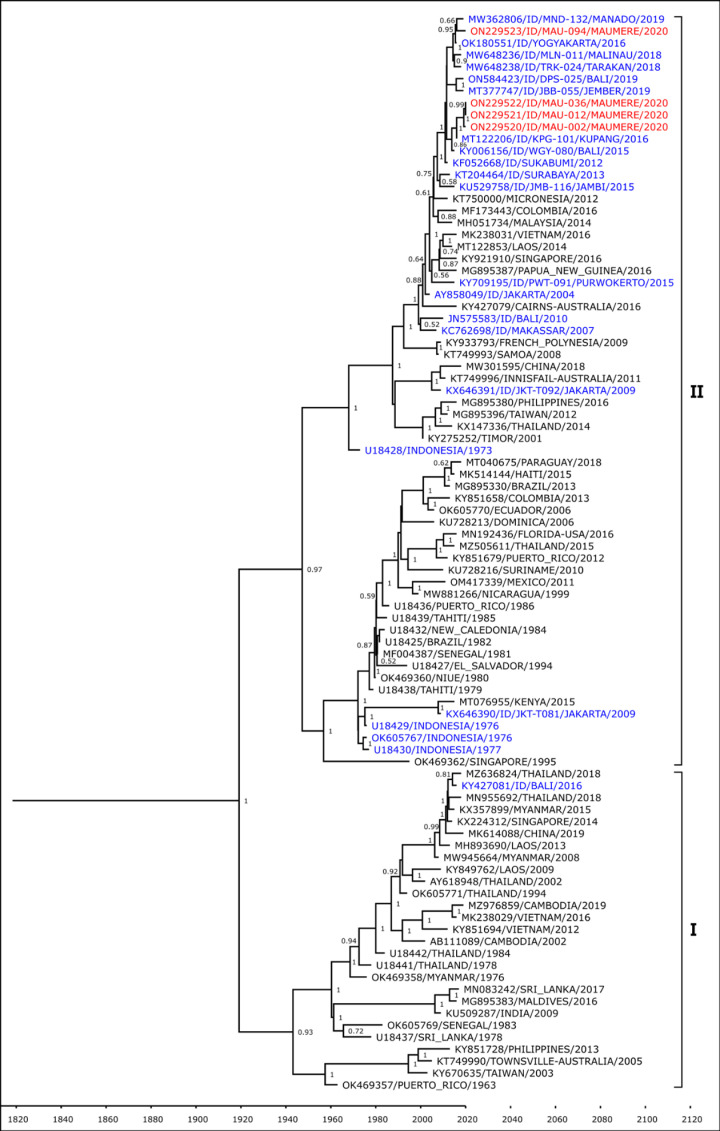
Phylogenetic analysis of Maumere DENV-4 (red font) together with closest strains from Indonesia (blue font) and strains from surrounding countries (black font).

Lastly, all four DENV-4 isolates were shown to belong to Genotype II based on Lanciotti’s classification [27] (Fig 6). The Maumere isolates formed two separate clades, one is related to DENV-4 strains from Indonesian cities of Jember in East Java, and the other clade consists of strains from Bali and Kupang, the capital of East Nusa Tenggara province.

## Discussion

Outbreak of a disease in regions with low endemicity warrants epidemiologic investigations. We investigated the molecular-epidemiological aspects of a severe dengue outbreak in Maumere and revealed that DENV-3 was the causative agent responsible for the outbreak. The severity of disease in this outbreak is reflected by most cases in this study being DHF and DSS, as well as the number and severity of cases throughout the municipality catching national media attention. Outbreaks of severe dengue have been reported previously in Indonesia since at least 1975 [32], and recently in Manado in 2019, where the predominant infecting serotype was also DENV-3 [33]. Furthermore, DENV-3 has been found to have a higher percentage of severe cases compared to other serotypes [34].

The combination of epidemiological survey with molecular and serological examination proves useful in assisting the health authority in understanding the etiology of the outbreak. In rural regions with limited resources like Maumere, epidemiological investigation conducted together with stake holders such as health authority, academics, and scientists will be beneficial to comprehensively understand the local health problems. To the authors’ knowledge, this study is the first to report clinical and virological data on dengue in the city of Maumere. The nearest cities in Indonesia where similar studies on dengue have been done are in Kupang in 2017 [8], Denpasar in 2015 [35], and Makassar in 2010 [36]. In those studies, the predominant serotype found in Kupang and Denpasar is the same as that in Maumere (DENV-3), while the predominant serotype in Makassar was DENV-1, and a large percentage of dengue cases in all three cities were secondary infections [8,35,36].

All four DENV serotypes were found circulating in Maumere, as they do in other Indonesian cities, though the predominant serotype differs between regions. In 2019, DENV-1 was the predominant serotype in Batam [37], DENV-2 was the predominant serotype in Ambon [37] and North Kalimantan [31], DENV-3 was the predominant serotype in Banjarmasin [37] and Manado [33], while DENV-4 was the predominant serotype in Jember [30]. Serotype shifts in a region are often associated with local outbreaks [38]. Unfortunately, there is no previous serotype data on dengue cases from Maumere with which comparisons could be made. Thus, our data provide important information of DENV serotypes circulation in the region which will be useful for future mitigation.

In this study, indirect IgG ELISA results which detect the presence of anti-dengue IgG in the patients demonstrate high level of past exposure of DENV even in young children. The high proportion of secondary infections in this study highly likely relates to the severity of the outbreak, possibly due to the antibody-dependent enhancement mechanism [39]. PRNT was performed on DENV-3 secondary infections to determine the titer of anti-dengue antibody according to the serotype of their past dengue infections. This is especially important to see which serotypes the patients have been exposed to previously. The PRNT results show most of these patients having neutralizing antibodies, though on different levels, to all four DENV serotypes. Median PRNT_50_ titer levels are highest in DENV-2, which may indicate a previous high exposure to DENV-2 circulating within the community/region, which then shifted to the current DENV-3 predominance (Fig 2C). In a previous study evaluating subsequent heterologous DENV infections in Thailand, DENV-3 post-primary infections that were preceded by DENV-2 infections were found to have a higher percentage of DHF cases compared to DF [40]. Furthermore, in general dengue infections in Maumere have higher AR compared to other regions in Indonesia during the previous year, suggesting a higher force of infection contributing to the outbreak.

Phylogenetic analyses show that DENV isolates from Maumere are closely related to those from Bali and Kupang. Travel may play a considerable factor in dengue transmission and introduction to a region as both these cities have direct flights to Maumere. Geographical proximity most likely also contributes to genetic similarities of DENV, as observed in the closeness of Maumere and Timor Leste strains in the phylogenetic trees. Maumere and Timor Leste share a land border, which facilitates frequent human movement between the two countries.

The DENV-1 isolate from Maumere belongs to the Genotype IV group along with 2007–2012 isolates from other Indonesian cities. Only two DENV-1 cases were detected in this study, with only one successfully sequenced. More recent (2014–2019) DENV-1 isolates from Indonesia belong to Genotype I, a group which has been observed to have a relatively higher rate of replication compared to Genotype IV [36], suggesting the possibility of clade replacement in those areas. The possibility of this clade replacement already happening in Maumere cannot be ruled out due to the limited number of DENV-1 cases detected in this study.

The predominant serotype found in Maumere was DENV-3, with all sequenced isolates grouped into Genotype I. Further sub-grouping within this genotype was observed between these Maumere isolates, with one sub-group having a lineage closer to isolates from Bali, while the other sub-group was closer to isolates from cities in Kalimantan. DENV genotype may be related to dengue severity as observed in previous studies, where infections with Southeast Asian genotype of DENV-2 is related to severe outbreak in 14 countries in Latin America [41]. However, the vast majority of DENV-3 found circulating in Indonesia belongs to the Genotype I group [6], which is also reflected in this outbreak in Maumere where all the DENV-3 sequenced belongs to the Genotype I group. Due to this, we are unable to determine whether there is a direct relationship between disease severity and DENV genotype in this study.

## Conclusion

This study reveals the etiology of the outbreak in Maumere in which most cases were caused by DENV-3 infection, within the background of the other three DENV serotypes circulation. The high percentage of anti-dengue antibodies for multiple serotypes suggests a history of dengue transmission with a high infection rate in the area. Introduction of a new strain of DENV into populations with low herd immunity against that introduced strain can cause outbreaks of severe dengue, highlighting the importance of serotype and genotype surveillance. This study provides new baseline information on the virologic, immunology, and genomic data on dengue in Maumere, East Nusa Tenggara. This study also demonstrates the importance of outbreak investigations and to inform health authorities’ policy-making on disease management/infectious control measures.

## Supporting information

S1 Fig Map of Indonesia (light blue) showing the geographical location of Maumere and other surrounding Indonesian cities and Timor Leste as the origin of DENV strains described in the phylogenetic trees. (PDF)

S1 Table PRNT_50_ titers of neutralizing antibodies for each DENV serotype. (PDF)
