# Supplementary material for: Investigation of severe dengue outbreak in Maumere, East Nusa Tenggara, Indonesia: Clinical, serological, and virological features
Source: PLoS One. 2025 Feb 18;20(2):e0317854. doi: 10.1371/journal.pone.0317854 (PMC11835340; doi:10.1371/journal.pone.0317854)
Supplement: S1 Fig — (PDF) [file pone.0317854.s001.pdf]

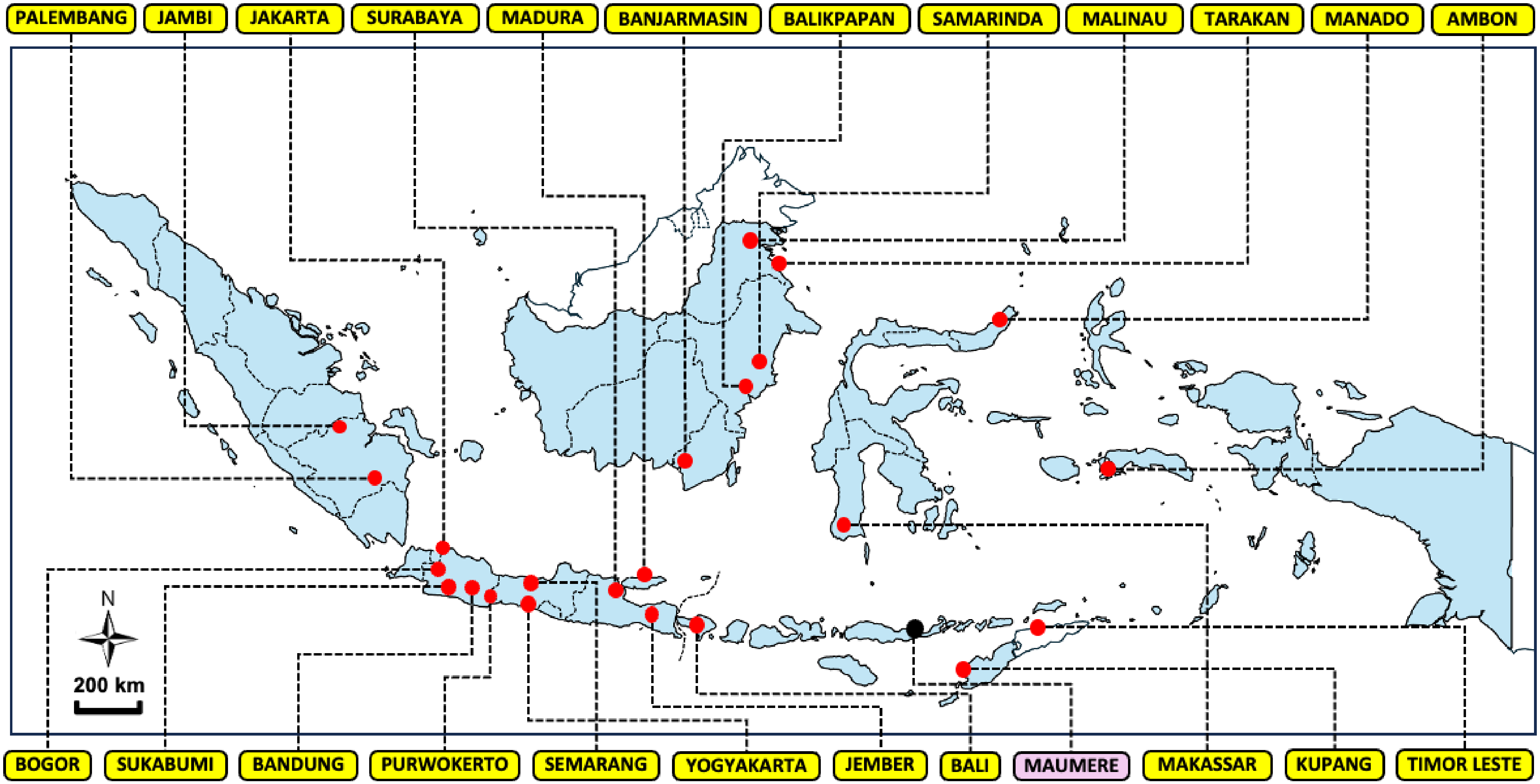

Supplementary Figure. Map of Indonesia (light blue) showing the geographical location of Maumere and other surrounding Indonesian cities and Timor Leste as the origin of DENV strains described in the phylogenetic trees.
