# Supplementary material for: Investigation of severe dengue outbreak in Maumere, East Nusa Tenggara, Indonesia: Clinical, serological, and virological features
Source: PLoS One. 2025 Feb 18;20(2):e0317854. doi: 10.1371/journal.pone.0317854 (PMC11835340; doi:10.1371/journal.pone.0317854)
Supplement: S1 Table — (PDF) [file pone.0317854.s002.pdf]

Supplementary Table 1. *$PRNT_{50}$ titers of neutralizing antibodies for each DENV serotype*

**Sample No.**	**PRNT Challenge Virus**			
	**DENV-1**	**DENV-2**	**DENV-3**	**DENV-4**
1	34	61	5209	19
2	277	3450	686	883
3	36	798	170	94
4	749	2053	4973	7673
5	158	1515	848	1148
6	98	1216	287	172
7	230	1077	1873	505
8	364	5936	4077	663
9	337	227	1606	1207
10	234	3313	2917	159
11	<10	339	20	<10
12	51	386	572	111
13	273	555	203	83
14	72	447	103	151
15	12	< 10	43	36
16	288	987	816	402
